# A Poly‐L‐Lactic Acid Formulation for Nasolabial Fold Correction: Handling, Tolerability and Effectiveness in a Prospective Observational Study

**DOI:** 10.1111/jocd.71251

**Published:** 2026-11-01

**Authors:** Thomas Dirschka, Marcus Kuchner, Lutz Schmitz

**Affiliations:** ^1^ CentroDerm Clinic Wuppertal Germany; ^2^ Faculty of Health University of Witten‐Herdecke Witten Germany; ^3^ Department of Dermatology, Venereology and Allergology Ruhr‐University Bochum Germany

**Keywords:** biostimulator, dermal filler, handling, nasolabial folds, PLLA, poly‐L‐lactic acid

## Abstract

**Background:**

Poly‐L‐lactic acid (PLLA) is a biostimulator used for nasolabial fold (NLF) correction. This study evaluated product handling, tolerability, and aesthetic outcomes.

**Objectives:**

To assess handling and applicability of a PLLA formulation, tolerability, and pre‐specified effectiveness end points for NLF correction.

**Methods:**

This prospective, open‐label, uncontrolled, single‐center observational study included adults with moderate‐to‐very severe NLFs receiving up to three treatment sessions, with follow‐up at 4, 12, and 26 weeks after the last injection. Handling was assessed using a 12‐item questionnaire. Effectiveness was assessed using MAS‐NLF, IGAIS, and SGAIS.

**Results:**

Of 35 screened individuals, 33 were treated and 21 (63.6%) were assessed at Week 26. Handling was rated favorably. Four participants (12.1%) experienced adverse events, including treatment‐related slight bilateral swelling in two (6.1%). No serious adverse events, device deficiencies, papules, nodules or granulomas were documented through Week 26. At Week 26, improvement of at least one MAS‐NLF grade was recorded in 4/21 participants (19.0%), IGAIS of at least “improved” in 14/21 (66.7%) and SGAIS in 11/21 (52.4%).

**Conclusions:**

Handling was rated favorably under the conditions studied, and descriptive improvements in NLF severity and global aesthetic ratings were observed. The uncontrolled design, sample size, and follow‐up limit conclusions regarding treatment‐specific effectiveness and uncommon or delayed adverse events.

**Trial Registration:**

German Clinical Trials Register (DRKS00032634; registered 6 September 2023)

## 1 Introduction

Poly‐L‐lactic acid (PLLA) is a biodegradable, biostimulatory filler that has been used in aesthetic medicine for more than two decades. Unlike hyaluronic acid fillers, which mainly provide immediate volumization, PLLA induces neocollagenesis through stimulation of fibroblasts, resulting in gradual and long‐lasting correction of soft‐tissue volume loss [1, 2, 3, 4, 5]. Clinical studies have demonstrated its effectiveness in treating facial lipoatrophy and deep wrinkles such as nasolabial folds (NLFs) [4, 5, 6, 7]. Beyond efficacy, prominent nasolabial folds carry psychosocial weight. Facial wrinkles bias first impressions and are associated with less favorable social evaluations, while minimally invasive treatments improve appearance‐related well‐being and quality of life [8, 9]. Patients also value results that are gradual and longer lasting, reducing retreatment burden [10].

Preparation and injection are integral to PLLA treatment, and technical considerations of reconstitution and injection have been addressed in the literature [11, 12].

Papules, nodules and late granulomas are recognized adverse events associated with PLLA, with reported rates varying between studies [13, 14, 15].

The product evaluated in the present study (Juläine, Nordberg Medical AB, Huddinge, Sweden) is a CE‐marked injectable implant indicated for augmentation of shallow to deep nasolabial folds. According to the manufacturer's instructions for use, it contains PLLA microspheres, sodium carboxymethylcellulose and mannitol [16].

The present prospective observational study was conducted to evaluate the handling and applicability of this PLLA formulation, its tolerability, and pre‐specified effectiveness endpoints for NLF correction.

## 2 Methods

### 2.1 Study Design and Patients

This was a prospective, open‐label, uncontrolled, single‐center observational study conducted at a single center in Germany. Recruitment of 35 participants was planned. No formal sample size calculation was performed.

Adults (≥ 18 years) with moderate‐to‐very severe NLFs (MAS‐NLF grades 2–4) were eligible [17]. Patients were required to abstain from additional facial interventions in the treatment area during the study period, except for botulinum toxin in nontarget regions. Key exclusion criteria included prior treatment of the NLF with PLLA, polymethylmethacrylate, calcium hydroxylapatite, silicone, or autologous fat. Further exclusion criteria were systemic immunosuppression, severe autoimmune disease, active malignancy, history of keloid formation, coagulation disorders, and pregnancy or lactation. Previous hyaluronic acid injections were permitted if administered ≥ 12 months before baseline. After screening, participants received a first treatment and, if considered necessary by the investigator, up to two additional treatments at intervals of 2–4 weeks. When no further treatment was required at the second or third treatment visit, the visit proceeded as the Week‐4 follow‐up. Follow‐up visits were scheduled 4, 12, and 26 weeks after the last injection.

### 2.2 Treatment Protocol

Each vial (150 mg PLLA, 45 mg sodium carboxymethylcellulose, 145 mg mannitol) was reconstituted with 5 mL 0.9% sodium chloride solution, yielding a final PLLA concentration of 30 mg/mL. The reconstituted product was left to stand for 2 h before use.

Injections were placed slightly medial to each NLF at a minimum of three cranial, middle, and caudal points using a subdermal bolus technique, with 0.1–0.2 mL per point at intervals of 0.5–1 cm and a maximum of 1.5 mL per side and 3.0 mL bilaterally per session. Aspiration was performed for at least 2 s before each injection. No local or topical anesthesia was used. Injections were performed with a 30‐gauge needle. The manufacturer's instructions for use specify a 26‐gauge needle [16]. The treated area was massaged for at least 2 min immediately after injection. Participants were instructed to continue massage for 5 min, 5 times daily, for 5 consecutive days and to avoid make‐up for 24 h and extreme heat or cold for 72 h.

### 2.3 Safety Assessments

Safety was monitored throughout the study based on investigator assessment and participant reporting. Adverse events, adverse device effects, and device deficiencies were recorded at each visit. Serious adverse events were to be reported within 24 h.

### 2.4 Handling Assessment

Immediately after each treatment session, the investigator who had performed the injection completed a study‐specific 12‐item questionnaire assessing preparation, injection, and postinjection handling on a 0–10 visual analogue scale (0 = do not agree at all, 10 = fully agree; Supplemental Digital Content 2 [Data S1]). A separate questionnaire was completed for each treatment session.

The questionnaire was not formally validated. Four items were negatively phrased (reconstitution effort, needle clogging, need for needle changes, and massage effort) and were analyzed as recorded without reverse coding. Lower ratings therefore indicate more favorable handling for these items. Ratings were unblinded, and no comparator product was assessed. Two investigators performed the treatments and assessments.

### 2.5 Effectiveness Assessments

The pre‐specified effectiveness endpoints at Week 26 were improvement of at least one MAS‐NLF grade, an IGAIS rating of at least “improved” (+1) and an SGAIS rating of at least “improved” (+1). The same assessments at earlier visits were pre‐specified secondary end points.

NLF severity was assessed using the validated 5‐point MAS‐NLF (0 = none; 4 = very severe) [17]. IGAIS and SGAIS assessed global change relative to baseline on a 5‐point scale from “worse” (−1) to “very much improved” (+3). IGAIS was rated by the investigator and SGAIS by the participant using baseline photographs; participants additionally used a mirror for SGAIS.

### 2.6 Statistical Analysis

Analyses were performed by an external statistician using IBM SPSS Statistics. The Safety Set comprised all participants who received at least one injection (*n* = 33). Effectiveness and handling analyses were based on observed cases with visit‐specific denominators. No imputation of missing data was performed.

Continuous variables were summarized descriptively and categorical variables as *n* (%). Questionnaire ratings were tested for normality using the Kolmogorov–Smirnov and Shapiro–Wilk tests and paired treatment‐session ratings were compared using the Wilcoxon signed‐rank test. Change from baseline was tested using the McNemar test where a symmetric contingency table was available. Tests were two‐sided at *α* = 0.05 without adjustment for multiplicity. Inferential analyses were exploratory and non‐confirmatory.

### 2.7 Ethics Statement

The study was reviewed and approved by the responsible ethics committee before study start and was conducted in accordance with the Declaration of Helsinki. All participants provided written informed consent, including consent for publication of de‐identified photographs. The study was registered in the German Clinical Trials Register (DRKS00032634; 6 September 2023).

## 3 Results

### 3.1 Patient Disposition and Baseline Characteristics

Of 35 screened individuals, 33 received at least one injection and constituted the safety population (Figure 1). Additional treatment sessions were administered according to investigator‐assessed need: 31 participants received a second and 26 a third treatment session. Follow‐up assessments were available for 28 participants at Week 4, 25 at Week 12 and 21 at Week 26 (21/33, 63.6%). Documented reasons for early study end were available for some, but not all participants.

**FIGURE 1:**
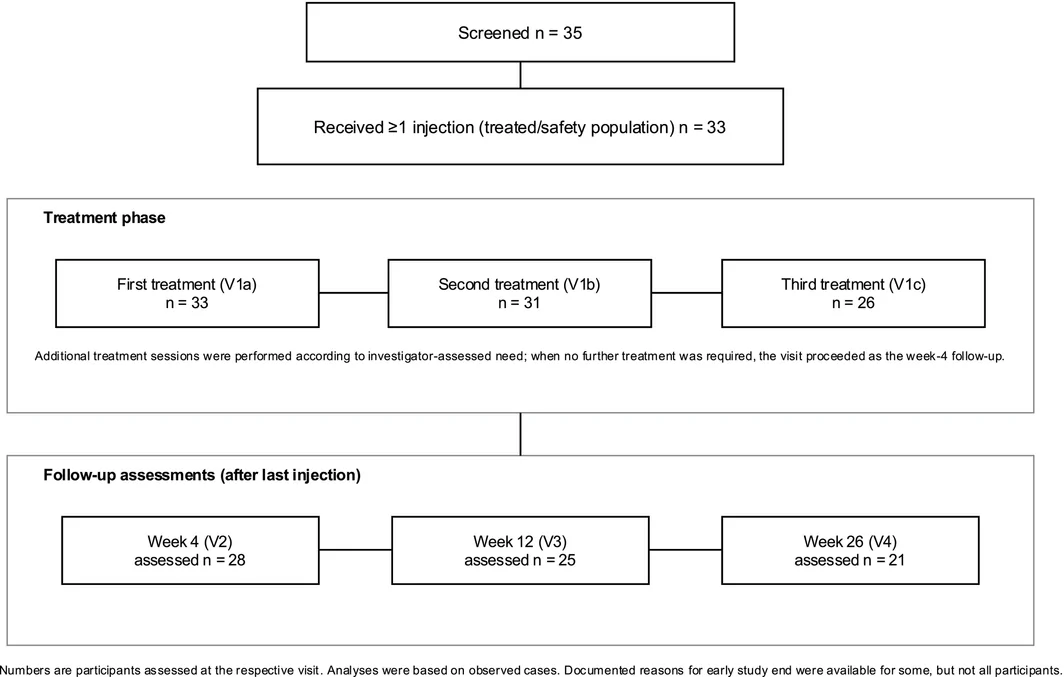
Participant flow. Thirty‐five individuals were screened and 33 received at least one injection (treated/safety population). Additional treatment sessions were performed according to investigator‐assessed need. When no further treatment was required at the second or third treatment visit, the visit proceeded as the Week‐4 follow‐up visit according to the study plan. Follow‐up assessments were available for 28 participants at Week 4, 25 at Week 12, and 21 at Week 26. Analyses were based on observed cases without imputation. Documented reasons for early study end were available for some, but not all participants.

Of the 33 treated participants, 28 (84.8%) were women and 5 (15.2%) were men. Mean age was 51.3 years (SD 12.1; range 32–76). At baseline, 22/33 participants (66.7%) had MAS‐NLF Grade 2 and 11/33 (33.3%) Grade 3.

### 3.2 Safety

Four adverse events were documented in four of 33 treated participants (12.1%; Table 1). Two participants (6.1%) experienced treatment‐related slight bilateral swelling in the treatment area, ending within 1 and 2 days, respectively. No intervention was required for either event. Atopic dermatitis and pollinosis with cat allergy occurred in one participant each and were considered unrelated to treatment. No serious adverse events or device deficiencies were documented. No papules, nodules, or granulomas were documented through Week 26.

**TABLE 1:** Adverse events in the safety population (*n* = 33). Each event occurred in one participant. No formal severity grading beyond the source wording was documented. No serious adverse events or device deficiencies were documented. No papules, nodules or granulomas were documented through Week 26.

Event	Participants, *n*	Relationship to treatment	Start date	End date/status
Slight swelling of the left and right side in the treatment area	1	Related	27 May 2024	28 May 2024; resolved
Slight swelling of the left and right side in the treatment area	1	Related	3 June 2024	5 June 2024; resolved
Atopic dermatitis	1	Not related	6 June 2024	Ongoing at last documentation
Pollinosis, cat allergy	1	Not related	10 April 2024	Ongoing at last documentation

### 3.3 Handling Assessment

Handling ratings were available for 33, 31, and 26 treatment sessions at the first, second, and third session, respectively, for nine questionnaire items and for 32, 30, and 26 sessions for items 2, 8, and 9. Complete item‐level *n*, mean, and SD values are provided in Supplemental Digital Content 3 (Data S1) and are shown graphically in Supplemental Digital Content 1 (Data S1). At the first treatment session, mean ratings ranged from 8.16 to 9.79 for positively phrased items and from 0.11 to 1.20 for negatively phrased items, for which lower values indicate more favorable handling. Of 24 exploratory paired comparisons with the first session, one reached the nominal 5% level (item 12, third session; *p* = 0.025). No adjustment for multiplicity was applied. No consistent change in handling across sessions was observed.

### 3.4 Effectiveness

Improvement of at least one MAS‐NLF grade was recorded in 1/31 participants (3.2%) at the second treatment session, 3/26 (11.5%) at the third treatment session, 5/28 (17.9%) at Week 4, 5/25 (20.0%) at Week 12 and 4/21 (19.0%) at Week 26. No participant was rated worse than baseline at any assessed visit (Table 2, Figure 2).

**TABLE 2:** Nasolabial fold severity by visit (MAS‐NLF) and improvement relative to baseline, based on participants assessed at each visit. Data are *n* (%) of participants assessed at the respective visit, unless stated as n/N (%). Week 4, Week 12, and Week 26 refer to the interval after the last injection. Baseline is the assessment made before the first injection. The pre‐specified effectiveness threshold was an improvement of at least one full MAS‐NLF grade relative to baseline. Analyses are based on observed cases without imputation. Denominators differ between visits because not all participants were assessed at every visit. Percentages may not sum to 100% due to rounding.

MAS‐NLF grade	Baseline *N* = 33	Second treatment *N* = 31	Third treatment *N* = 26	Week 4 *N* = 28	Week 12 *N* = 25	Week 26 *N* = 21
0—none	0 (0)	0 (0)	0 (0)	0 (0)	0 (0)	0 (0)
1—mild	0 (0)	0 (0)	1 (3.8)	2 (7.1)	3 (12.0)	2 (9.5)
2—moderate	22 (66.7)	21 (67.7)	17 (65.4)	20 (71.4)	17 (68.0)	14 (66.7)
3—severe	11 (33.3)	10 (32.3)	8 (30.8)	6 (21.4)	5 (20.0)	5 (23.8)
4—very severe	0 (0)	0 (0)	0 (0)	0 (0)	0 (0)	0 (0)
Improvement of ≥ 1 grade versus baseline	—	1/31 (3.2)	3/26 (11.5)	5/28 (17.9)	5/25 (20.0)	4/21 (19.0)
Worse than baseline	—	0/31 (0)	0/26 (0)	0/28 (0)	0/25 (0)	0/21 (0)

Abbreviation: MAS‐NLF, Merz Aesthetics Nasolabial Fold Severity Scale.

**FIGURE 2:**
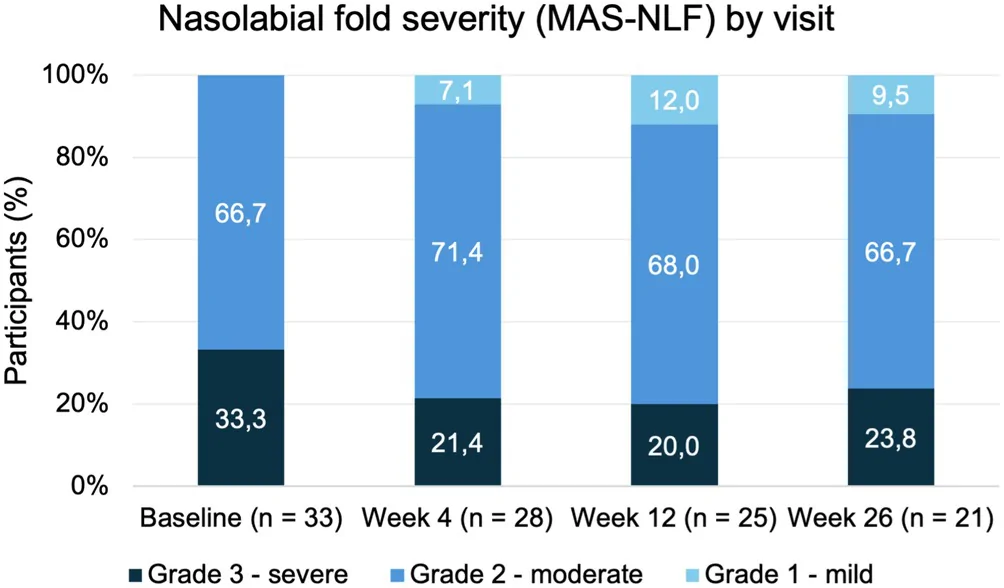
Nasolabial fold severity by visit. Distribution of Merz Aesthetics Nasolabial Fold Severity Scale (MAS‐NLF) grades among participants assessed at baseline and at weeks 4, 12, and 26 after the last injection. Bars show observed‐case percentages; denominators are shown with the visit labels. Grades 0 and 4 were not observed at the visits shown. Complete category distributions, including the second and third treatment sessions, are provided in Table 2. Percentages may not sum to 100% due to rounding.

IGAIS of at least “improved” was recorded in 5/31 participants (16.1%), 17/26 (65.4%), 18/28 (64.3%), 17/25 (68.0%) and 14/21 (66.7%) at the corresponding visits. SGAIS rates were 8/31 (25.8%), 16/26 (61.5%), 16/28 (57.1%), 15/25 (60.0%) and 11/21 (52.4%), respectively (Table 3, Figure 3). One participant reported “worse” on the SGAIS at Week 4. All analyses were based on observed cases without imputation.

**TABLE 3:** Data are *n* (%) of participants assessed at the respective visit, unless stated as *n*/*N* (%). Both scales record global change relative to baseline. IGAIS ratings were made by the investigator and SGAIS ratings by the participant. Week 4, Week 12, and Week 26 refer to the interval after the last injection. The pre‐specified effectiveness threshold was a rating of at least “improved” (+1). Analyses are based on observed cases without imputation. Denominators differ between visits because not all participants were assessed at every visit. Percentages may not sum to 100% due to rounding.

Rating	Second treatment *N* = 31	Third treatment *N* = 26	Week 4 *N* = 28	Week 12 *N* = 25	Week 26 *N* = 21
IGAIS					
Very much improved (+3)	0 (0)	0 (0)	1 (3.6)	0 (0)	0 (0)
Much improved (+2)	0 (0)	0 (0)	1 (3.6)	0 (0)	0 (0)
Improved (+1)	5 (16.1)	17 (65.4)	16 (57.1)	17 (68.0)	14 (66.7)
No change (0)	26 (83.9)	9 (34.6)	10 (35.7)	8 (32.0)	7 (33.3)
Worse (−1)	0 (0)	0 (0)	0 (0)	0 (0)	0 (0)
Rating ≥ improved (+1)	5/31 (16.1)	17/26 (65.4)	18/28 (64.3)	17/25 (68.0)	14/21 (66.7)
SGAIS					
Very much improved (+3)	0 (0)	0 (0)	1 (3.6)	1 (4.0)	0 (0)
Much improved (+2)	0 (0)	2 (7.7)	1 (3.6)	2 (8.0)	0 (0)
Improved (+1)	8 (25.8)	14 (53.8)	14 (50.0)	12 (48.0)	11 (52.4)
No change (0)	23 (74.2)	10 (38.5)	11 (39.3)	10 (40.0)	10 (47.6)
Worse (−1)	0 (0)	0 (0)	1 (3.6)	0 (0)	0 (0)
Rating ≥ improved (+1)	8/31 (25.8)	16/26 (61.5)	16/28 (57.1)	15/25 (60.0)	11/21 (52.4)

Abbreviations: IGAIS, Investigator Global Aesthetic Improvement Scale; SGAIS, Subject Global Aesthetic Improvement Scale.

**FIGURE 3:**
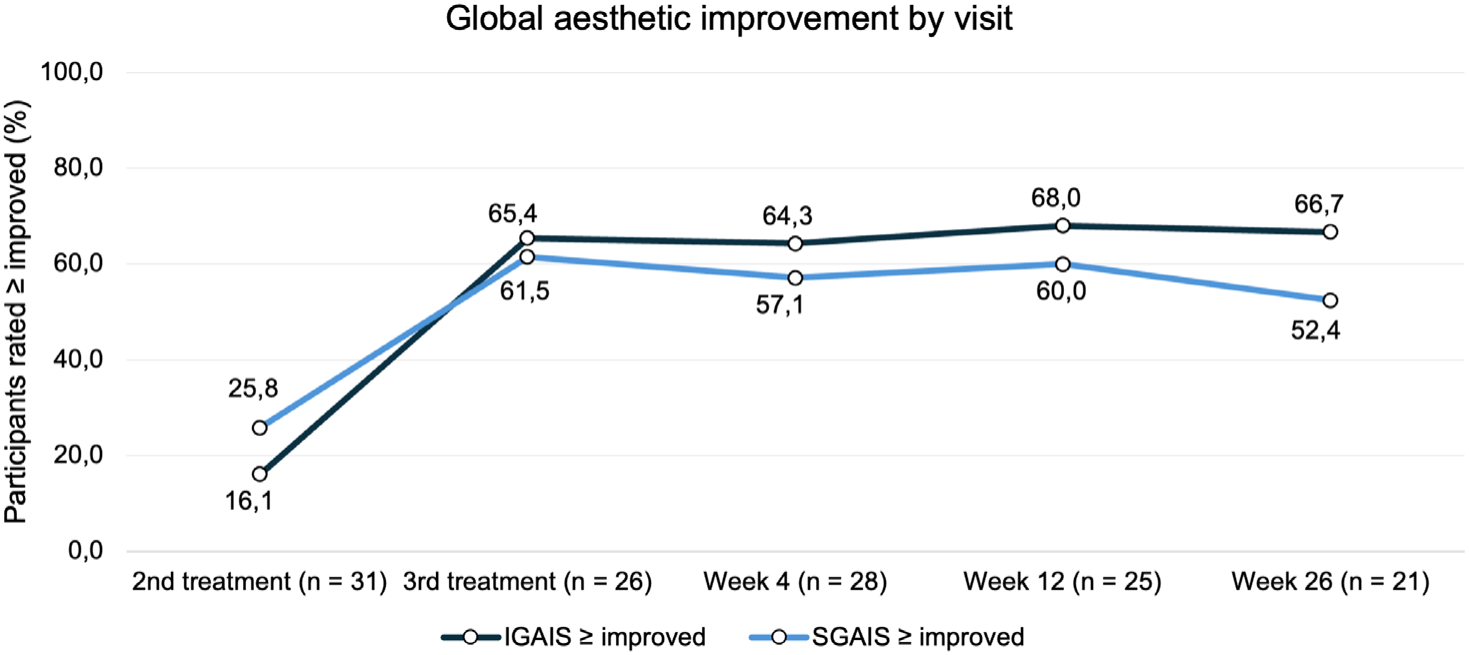
Global aesthetic improvement by visit. Proportions of participants rated at least “improved” (+1) on the Investigator Global Aesthetic Improvement Scale (IGAIS) and Subject Global Aesthetic Improvement Scale (SGAIS) at each assessed visit. Points represent observed‐case proportions at the respective visit. Denominators are shown with the visit labels. Week 4, Week 12, and Week 26 refer to the time after the last injection. Complete category distributions for IGAIS and SGAIS are provided in Table 3.

## 4 Discussion

This prospective observational study evaluated the handling, tolerability and pre‐specified effectiveness end points of a PLLA formulation for NLF correction. Handling ratings were predominantly favorable under the conditions studied. Four adverse events occurred in four of 33 treated participants, including two short‐lived treatment‐related events of bilateral swelling. At Week 26, 4/21 participants (19.0%) had improved by at least one MAS‐NLF grade, while 14/21 (66.7%) and 11/21 (52.4%) were rated at least “improved” on IGAIS and SGAIS, respectively.

Handling was assessed immediately after treatment by the injecting investigator using a study‐specific 12‐item questionnaire. Ratings were predominantly at the favorable end of the scale for both positively and negatively phrased items. The instrument was not formally validated; ratings were unblinded, and no comparator product was assessed. Notably, these ratings were obtained using the 30‐gauge needle actually used in the study, whereas the manufacturer's instructions for use specify a 26‐gauge needle. The findings therefore provide a descriptive account of handling under the conditions studied rather than a comparison with other PLLA products.

The proportions meeting the pre‐specified effectiveness thresholds differed between MAS‐NLF and the global improvement scales. These measures assess different aspects of aesthetic change: the MAS‐NLF end point required improvement of at least one full severity grade, whereas IGAIS and SGAIS assessed global change relative to baseline. In the absence of a control group and blinded independent assessment, the magnitude of any treatment‐specific effect cannot be determined. Clinical effects of PLLA have been described as developing gradually over weeks to months, consistent with its biostimulatory mechanism [18].

No papules, nodules, or granulomas were documented through Week 26. These events are recognized adverse events associated with PLLA, with reported rates varying between studies [13, 14, 15]. However, the small cohort, 63.6% Week‐26 attendance, and limited follow‐up preclude conclusions regarding the incidence of uncommon or delayed events. The manufacturer's instructions for use state that nodules, late granulomas and subcutaneous papules may occur up to 14 months after injection [16].

The principal limitations are the single‐centre, open‐label, and uncontrolled design, absence of blinded independent outcome assessment, small sample size, and observed‐case analysis with 36.4% attrition by Week 26. Selective loss to follow‐up therefore cannot be excluded and may have biased both safety and effectiveness estimates. The handling questionnaire was not formally validated, and no comparator product was assessed. Clinical photography was not fully reproducible between visits with respect to camera‐to‐subject distance, head positioning, hair positioning, and lighting and should therefore be regarded as illustrative (Figure 4). Finally, although eligibility permitted MAS‐NLF grades 2–4, all treated participants had Grade 2 or 3 NLFs at baseline, limiting generalisability to very severe NLFs. Strengths include prospective data collection, pre‐specified endpoints, and systematic adverse‐event recording.

**FIGURE 4:**
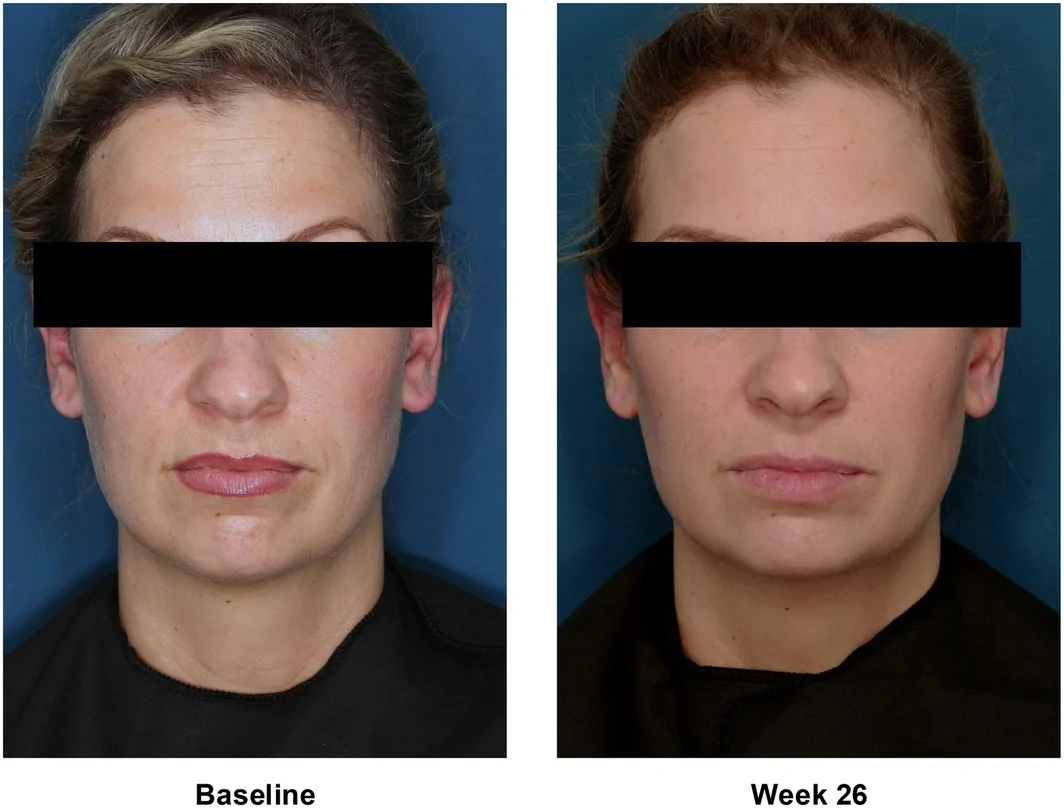
Clinical photographs of one participant. Left: Baseline, before the first injection. Right: Week 26, 26 weeks after the last injection. Photography was not fully standardized across visits with respect to camera‐to‐subject distance, head positioning, hair positioning, and lighting; the images are illustrative and do not constitute an independent measure of treatment effectiveness.

In summary, handling of this PLLA formulation was rated favorably under the conditions studied, and descriptive improvements in NLF severity and global aesthetic ratings were observed through Week 26. Two participants experienced short‐lived treatment‐related bilateral swelling, while no serious adverse events, device deficiencies, papules, nodules, or granulomas were documented. The uncontrolled design, sample size, and follow‐up limit conclusions regarding treatment‐specific effectiveness and uncommon or delayed adverse events.

## Funding

Funding and product supply. This study was funded by Nordberg Medical AB (Huddinge, Sweden), which also supplied the study product. Nordberg Medical AB had no role in the study design, data collection, statistical analysis, manuscript preparation, or the decision to submit the manuscript for publication.

## Ethics Statement

The study was reviewed and approved by the Ethics Committee of Witten/Herdecke University (approval no. 174/2023; 24 August 2023) and was conducted in accordance with the Declaration of Helsinki. All participants provided written informed consent, including consent for publication of de‐identified photographs.

## Conflicts of Interest

**L. Schmitz:** Advisory board member for Almirall Hermal and Pierre‐Fabre; consultant for Damae and Nordberg Medical; honoraria for lectures from Almirall Hermal, Biofrontera, Galderma, Mylan, Sanofi‐Aventis, Damae, Emblation, Speclipse, Infectopharm, ISDIN, La Roche‐Posay, Schulze & Böhm GmbH, SciBase. **M. Kuchner:** Advisory board member for Almirall, honoraria for lectures from AbbVie. **T. Dirschka:** Advisory board member for Almirall, Biofrontera, Infectopharm, LEO, Meda, Novartis, Riemser, Janssen; consultant for Almirall, Biofrontera, GSK, Dr. Pfleger, Galderma, Janssen‐Cilag, LEO, Mylan, NeraCare, Novartis, Pierre‐Fabre, Scibase, Smartinmedia AG, Vichy, Damae, Speclipse, Nordberg Medical; research grants from Almirall, Biofrontera, Galderma, ISDIN, La Roche‐Posay, Mylan, NeraCare, Schulze & Böhm GmbH, Smartinmedia AG, Vichy, Nordberg Medical; honoraria for lectures from Aesclepion, Almirall, Biofrontera, Dr. August Wolff, Galderma, GSK, Infectopharm, Janssen‐Cilag, LEO, Mylan, NeraCare, Novartis, Pierre‐Fabre, Pfizer, Riemser, UCB, Damae, Emblation, Speclipse. We have categorized conflicts of interest according to the following scheme and reported them in the conflict of interest form accordingly: A, Speaker honoraria; B, Advisory board member; C, Research grants; D, Consultant.

## Supporting information


**Data S1:** Supplemental Digital Content 1: Physician questionnaire ratings at injection visits (V1a–V1c). Bars represent mean ratings on a 0–10 visual analogue scale (VAS; 0 = do not agree at all, 10 = fully agree). Error bars indicate mean ± SD, truncated at the scale limits (0 and 10).
Supplemental Digital Content 2: Study‐specific handling questionnaire translated into English. The questionnaire was administered in German and has not been formally validated.
Supplemental Digital Content 3: Item‐level handling questionnaire ratings by treatment session. Values are based on the descriptive statistics. Ratings were made on a 0–10 visual analogue scale (VAS; 0 = do not agree at all, 10 = fully agree). Items 2, 8, 9 and 12 are negatively phrased and were analyzed as recorded without reverse coding. Lower ratings indicate more favorable handling for these items. For all other items, higher ratings indicate more favorable handling. Values are rounded to two decimal places.

## Data Availability

The data that support the findings of this study are available from the corresponding author upon reasonable request. Due to the inclusion of sensitive patient information, the data are not publicly available.
