# Supplementary material for: A Poly‐L‐Lactic Acid Formulation for Nasolabial Fold Correction: Handling, Tolerability and Effectiveness in a Prospective Observational Study
Source: J Cosmet Dermatol. 2026 Nov 1;25(11):e71251. doi: 10.1111/jocd.71251 (PMC13724798; doi:10.1111/jocd.71251)
Supplement: Supplementary file 1 [file JOCD-25-e71251-s001.docx]

### **Supplemental Digital Content 1**

Supplemental Digital Content 1: Physician questionnaire ratings at injection visits (V1a–V1c). Bars represent mean ratings on a 0–10 visual analogue scale (VAS; 0 = do not agree at all, 10 = fully agree). Error bars indicate mean ± SD, truncated at the scale limits (0 and 10).

**Supplemental Digital Content 2**

Supplemental Digital Content 2: Study-specific handling questionnaire translated into English. The questionnaire was administered in German and has not been formally validated.

1. The reconstitution time is short.

2. The reconstitution until the final solution is obtained is laborious.

3. Drawing up the syringe is easy and requires little pressure.

4. No air bubbles are formed during drawing up.

5. The injection pressure into the tissue is low.

6. The product can be injected easily.

7. The injection can be performed with continuous pressure, without major resistance during administration.

8. The needle clogs easily during treatment.

9. The needle needs to be changed frequently during treatment.

10. The application of Juläine is easy.

11. The application of Juläine is quick.

12. Massaging the injection sites afterwards is laborious.

**Supplemental Digital Content 3**

**Item-level handling questionnaire ratings by treatment session**

Values are based on the descriptive statistics. Ratings were made on a 0–10 visual analogue scale (VAS; 0 = do not agree at all, 10 = fully agree).

| **Item** | **Questionnaire item** | **V1a, n** | **V1a, mean ± SD** | **V1b, n** | **V1b, mean ± SD** | **V1c, n** | **V1c, mean ± SD** |
| --- | --- | --- | --- | --- | --- | --- | --- |
| 1 | Reconstitution time is short | 33 | 8.16 ± 1.32 | 31 | 8.46 ± 0.85 | 26 | 8.63 ± 0.90 |
| 2 | Reconstitution until the final solution is obtained is laborious | 32 | 1.20 ± 1.38 | 30 | 1.01 ± 1.68 | 26 | 1.13 ± 1.73 |
| 3 | Drawing up the syringe is easy and requires little pressure | 33 | 9.76 ± 0.50 | 31 | 9.68 ± 0.53 | 26 | 9.73 ± 0.45 |
| 4 | No air bubbles form while drawing up the syringe | 33 | 9.79 ± 0.41 | 31 | 9.81 ± 0.40 | 26 | 9.89 ± 0.32 |
| 5 | Injection pressure into the tissue is low | 33 | 9.31 ± 0.81 | 31 | 9.30 ± 0.86 | 26 | 9.20 ± 0.69 |
| 6 | The product is easy to inject | 33 | 9.46 ± 0.83 | 31 | 9.52 ± 0.76 | 26 | 9.50 ± 0.81 |
| 7 | Injection can be performed with continuous pressure without major resistance | 33 | 9.19 ± 0.80 | 31 | 9.13 ± 0.95 | 26 | 9.28 ± 1.15 |
| 8 | The needle clogs easily during treatment | 32 | 0.17 ± 0.51 | 30 | 0.38 ± 1.13 | 26 | 0.05 ± 0.19 |
| 9 | The needle needs to be changed frequently during treatment | 32 | 0.11 ± 0.53 | 30 | 0.28 ± 1.01 | 26 | 0.02 ± 0.01 |
| 10 | Application of Juläine is easy | 33 | 9.49 ± 0.56 | 31 | 9.52 ± 0.67 | 26 | 9.47 ± 0.64 |
| 11 | Application of Juläine is quick | 33 | 9.07 ± 0.82 | 31 | 9.27 ± 0.63 | 26 | 9.35 ± 0.68 |
| 12 | Subsequent massage of the injection sites is laborious | 33 | 0.53 ± 0.83 | 31 | 0.34 ± 0.70 | 26 | 0.17 ± 0.37 |

Items 2, 8, 9 and 12 are negatively phrased and were analysed as recorded without reverse coding. Lower ratings indicate more favourable handling for these items. For all other items, higher ratings indicate more favourable handling. Values are rounded to two decimal places.
